# HIF-1α induces the epithelial-mesenchymal transition in gastric cancer stem cells through the Snail pathway

**DOI:** 10.18632/oncotarget.14484

**Published:** 2017-01-04

**Authors:** Shi-wei Yang, Zhi-gang Zhang, Ying-xue Hao, Yong-liang Zhao, Feng Qian, Yan Shi, Ping-ang Li, Chun-yang Liu, Pei-wu Yu

**Affiliations:** ^1^ Department of General Surgery and Center of Minimal Invasive Gastrointestinal Surgery, Southwest Hospital, Third Military Medical University, Chongqing 400038, China; ^*^ These authors contributed equally to this work

**Keywords:** cancer stem cells (CSCs), epithelial–mesenchymal transition (EMT), hypoxia inducible factor-1α (HIF-1α), Snail

## Abstract

Substantial evidence suggests that the epithelial-mesenchymal transition (EMT) phenotype is associated with the invasive characteristics of cancer stem cells (CSCs),which possess an EMT phenotype that may predominate in tumor invasion and metastasis. However, the mechanisms for the generation and regulation of these CSCs have not been clearly defined. As hypoxia and EMT-related factors may have important functions in EMT-like CSCs, the aim of this study was to investigate the effects of hypoxia on these cells. CSCs were established from the gastric cancer cell lines MGC-803 and SGC7901, and the relationship between hypoxia and EMT-like CSCs was investigated in gastric cancer. After hypoxia treatment, some gastric CSCs exhibited a marked increase in hypoxia-inducible factor-1α (HIF-1α)expression and increased migration and invasion capabilities compared with the normoxic control. These CSCs were defined by activation of the mesenchymal cell marker Vimentin and by inhibition of the epithelial cell marker E-cadherin. Our analyses also show that HIF-1α was responsible for activating EMT via increased expression of the transcription factor Snail in gastric CSCs. Moreover, inhibition of Snail by shRNA reduced HIF-1α-induced EMT in gastric CSCs. The results demonstrated that hypoxia-induced EMT-like CSCs rely on HIF-1αto activate Snail, which may result in recurrence and metastasis of gastric cancer.

## INTRODUCTION

Gastric cancer iscurrentlythe fourth most common cancer and the second most common cause of cancer death worldwide. Despite the development of surgical techniques and chemotherapy, the five-year survival rate remains low due to metastasis, recurrence and multiple drug resistance [1]. In 1997, Bonnet and Dick reported increased tumorigenicity in immune deficient miceofCD34+ CD38−cells from patients with acute myeloid leukemia(AML).This was the first study to describe cancer stem cells [2], which have since been identified in various types of solid tumors [3–5].

The cancer stem cell theory hypothesizes the following: most cancer cells have a limited proliferative ability, and only a few tumor cells are capable of forming a new tumor. These self-renewing cells are called cancer stem cells (CSCs) [2]. Based on recent studies, CSCs are assumed to be responsible for metastasis and resistance to commonly used chemotherapy and radiotherapy regimens. Thus, targeting CSCs might represent a novel approach form proving patient outcomes [6]. Previous reports have shown that normal and cancer stem cells from neural and epithelial organs can be expanded as sphere-like cellular aggregates in serum-free medium containing epidermal growth factor (EGF) and basic fibroblast growth factor (bFGF) [7]. In this medium, the floating cells grow as three-dimensional spheroid clusters termed spheroid bodies or spheroid cells.

The epithelial-mesenchymal transition (EMT) was first described as a developmental process during which epithelial cells acquire a motile mesenchymal phenotype. EMT is now recognized as a critical event during carcinoma metastasis. Indeed, this process results in degradation of the surrounding matrix, which leads to invasion and intravasation and facilitates the reestablishment of cancer cell colonies at distant sites [8]. Recent studies have suggested that CSCs intrinsically possess characteristics that are associated with mesenchymal cells, with critical functionsin tumor initiation, growth, and metastasis [9]. Brabletz [10] observed that certain cancer cells in the tumor-host interface display an EMT phenotype and CSCs properties and hypothesized that those cells derive from stationary CSCs via acquisition of stem cell properties by EMT, underlying the invasion and metastasis of tumors. This subset of cells was termed migrating CSCs or EMT-like CSCs. Unfortunately, little is known about the regulatory mechanisms of these cells. The microenvironment of most solid tumors is hypoxic, and HIF-1α, which is the most important hypoxia-induced transcription factor, has multiple functions in tumor progression, including changes in the aggressive behavior of the tumor [11]. In addition, the activity of several EMT-related transcription factors, such as Twist1, Notch, VEGF, PDGF, Snail, TGF-β, and ZEB1, is controlled either directly or indirectly by hypoxia [12]. Therefore, a possible link between EMT-like CSCs and hypoxia has been suggested.

Snail, a zinc-finger transcription factor, has a crucial function in tumor progression by facilitating tumor cell migration and invasion [13]. Hypoxia may increase Snail expression and result in EMT in ovarian cancer cells [14] Moreover, HIF-1α possibly mediates repression of E-cadherin expression by up-regulating E-cadherin-specific repressors such as Snail [15]. Overall, hypoxia/HIF-1α may regulate Snail expression, leading to the induction of EMT-like CSCs.

In the present study, to enrich gastric CSCs, we cultured gastric cancer cells in serum-free medium with EGF and bFGF as described in the Materials and Methods section. We prepared spheroid body-forming cells in gastric cancer cell lines and determined whether these spheroid cells could acquire CSC characteristics. We then investigated the mechanisms of hypoxia-induced EMT-like CSCs in gastric CSCs through Snail activation.

## RESULTS

### Spheroid cell formation by parental gastric cancer cells

**Figure 1:**
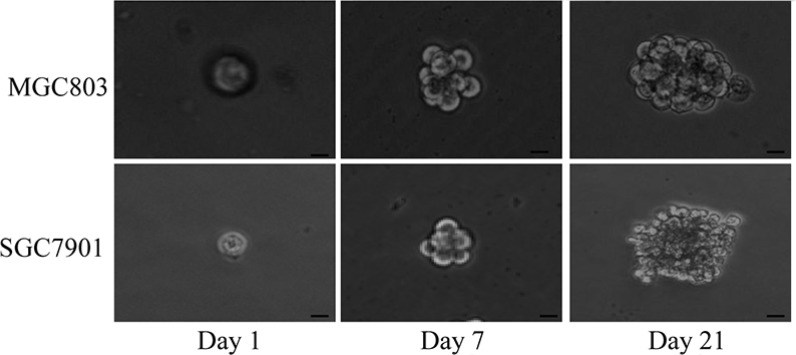
MGC803 and SGC7901 gastric cancer cells formed floating, self-renewing spheroid bodies Generation of a spheroid body from single MGC803 and SGC7901 cells on days 1, 7 and 21 in 96-well ultra-low-attachment dishes (200× magnification).

Previous reports have shown that both normal and CSCs from neural and epithelial organs can be expanded as sphere-like cellular aggregates in serum-free medium containing EGF and bFGF [16]. In the present study, we cultured MGC803 and SGC7901 gastric cancer cells in serum-free medium containing these growth factors. To assess their self-renewing capacity, we dissociated the spheroid bodies into single cells and grew them in serum-free medium in a 96-well ultra-low-attachment dish. Spheroid cells were observed in the medium7 d later, and spheroid bodies were completely formed after 21 d (Figure 1).

### Spheroid gastric cancer cells are enriched for gastric CSCs

**Figure 2:**
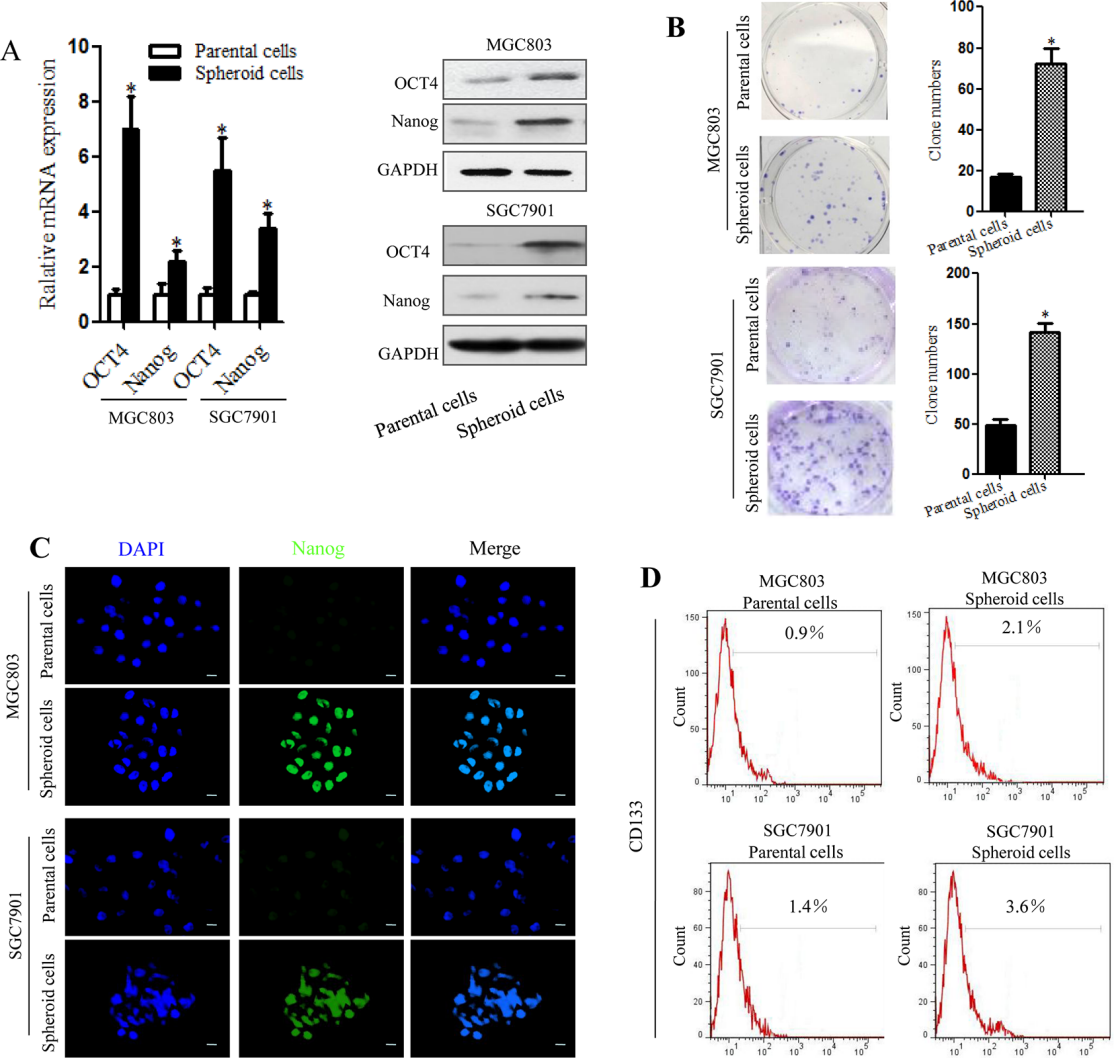
Spheroid cells derived from MGC803 and SGC7901cells have a high frequency of clone formation and overexpress gastric CSC-related genes (**A**) Expression of Oct 4 and Nanog mRNA and proteins, as detected by RT-PCR and western blotting, in MGC803 and SGC7901 spheroid cells and parental cells. (**B**) Clone formation by MGC803 and SGC7901 spheroid cells and parental cells. Quantitative analysis of the clone formation ability of MGC803 and SGC7901spheroid cells and parental cells. (**C**) Images of Nanog (green) protein expression, as detected by immunofluorescence staining; DAPI was used to stain nuclei. (**D**) Detection of CD133 expression by flow cytometry.*P < 0.05, three separate experiments with the same results were performed; error bars indicate SD.

To examine whether spheroid cells from gastric cancer cell lines are enriched with gastric CSCs, we compared several characteristics of these cells and the parental cells using quantitative real-time PCR analyses and western blotting. The results showed significantly increased expression of the CSC-related genes Oct4 and Nanog in spheroid cells than in parental cells (Figure 2A). Based on a colony formation assay, we also found higher colony formation efficiency of MGC803 or SGC7901spheroid cells compared to the parental cells (Figure 2B). Immunofluorescence staining confirmed the observed higher expression of Nanog in spheroid cells (Figure 2C).We also assessed CD133, a common CSC marker. As expected, MGC803 and SGC7901 spheroid cells expressed high levels of CD133, with CD133 positivity in 2.1% of MGC803 and 3.6% of SGC7901 spheroid cells. In contrast, only 0.9% of MGC803 and 1.4% of SGC7901 parental cells were CD133 positive (Figure 2D).

### *In vivo* tumorigenicity experiments

**Figure 3:**
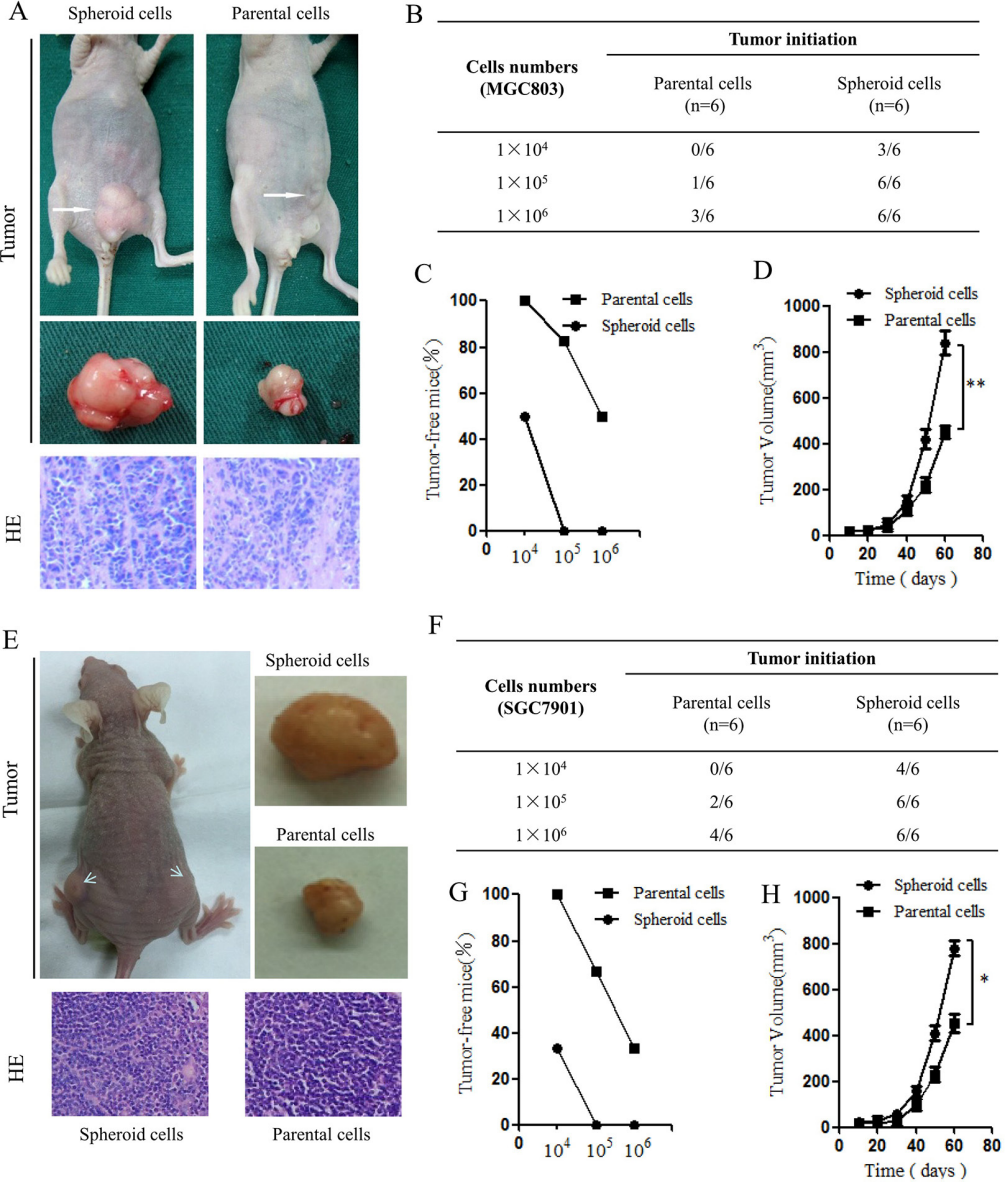
Tumorigenic capacity of MGC803 spheroid cells innude mice (**A**) Representative examples of xenograft tumors formed after subcutaneous injection of nude mice with 1 × 10^6^ MGC803 spheroid cells and parental cells. The representative xenograft tumor resulting from injection of spherical cells is larger in volume compared with that resulting from parental cell injection. H&E staining showing the histological features of the xenograft tumors. (**B**) Tumorigenicity of MGC803 spheroid cells compared with parental cells. (**C**) Ratios of tumor-free mice after injection of increasing numbers of MGC803 parental and spheroid cells after tumor formation for two months. *n* = 6 mice for each group. (**D**) Tumor-volume curves of MGC803 parental and spheroid cells injected into BALB/c nude mice. *n* = 6 mice. **P < 0.01. (**E**) Representative examples of xenograft tumors and H&E staining of SGC7901 spheroid cells and parental cells. (**F**) Tumorigenicity of SGC7901 spheroidcells compared with parental cells. (**G**) Ratios of tumor-free mice after injection of increasing numbers of SGC7901 parental and spheroid cells after tumor formation for two months. *n* = 6 mice for each group. (**H**) Tumor-volume curves of SGC7901 parental and spheroid cells injected into BALB/c nude mice. *n* = 6 mice. *P < 0.05.

Implanted tumors were harvested and fixed in formalin, and paraffin sections were cut and stained with hematoxylin and eosin (H&E). The volumes and weights of the transplanted tumors were also evaluated. Spheroid cells generated subcutaneous tumors with a larger volume compared to those generated from parental cells. H&E staining of the tumors showed that xenografts from spheroid cells had large nuclei and prominent nucleoli compared with xenografts from parental cells (Figure 3A). MGC803 spheroid cell generated 15/18 xenograft tumors, where as MGC803 parental cells generated 4/18 xenograft tumors. The xenograft formation proportions were as follows: spheroid cells (1 × 10^4^ cells: 3/6; 1 × 10^5^ cells: 6/6; and 1 × 10^6^ cells: 6/6) and parental cells (1 × 10^4^ cells: 0/6; 1 × 10^5^ cells: 1/6; and 1 × 10^6^ cells: 3/6). As few as 1 × 10^4^ spheroid cells were able to form xenograft tumors in nude mice (Figure 3B). Furthermore, the parental cells displayed much weaker tumor initiation and tumorigenic cell frequency, as assayed using a limiting dilution xenograft analysis (Figure 3C). According to the measured tumor volumes, the spheroid cells substantially enhanced tumor propagation compared with the parental cells (Figure 3D). The SGC7901 spheroid cells also showed higher tumorigenicity compared with the parental cells (Figure 3E–3H). Together, these data indicate that the spheroid cell subpopulations of gastric cell lines MGC803 and SGC7901 were enriched for gastric CSCs and exhibited higher tumorigenicity *in vivo*.

### Hypoxia-induced EMT-like CSCs

**Figure 4:**
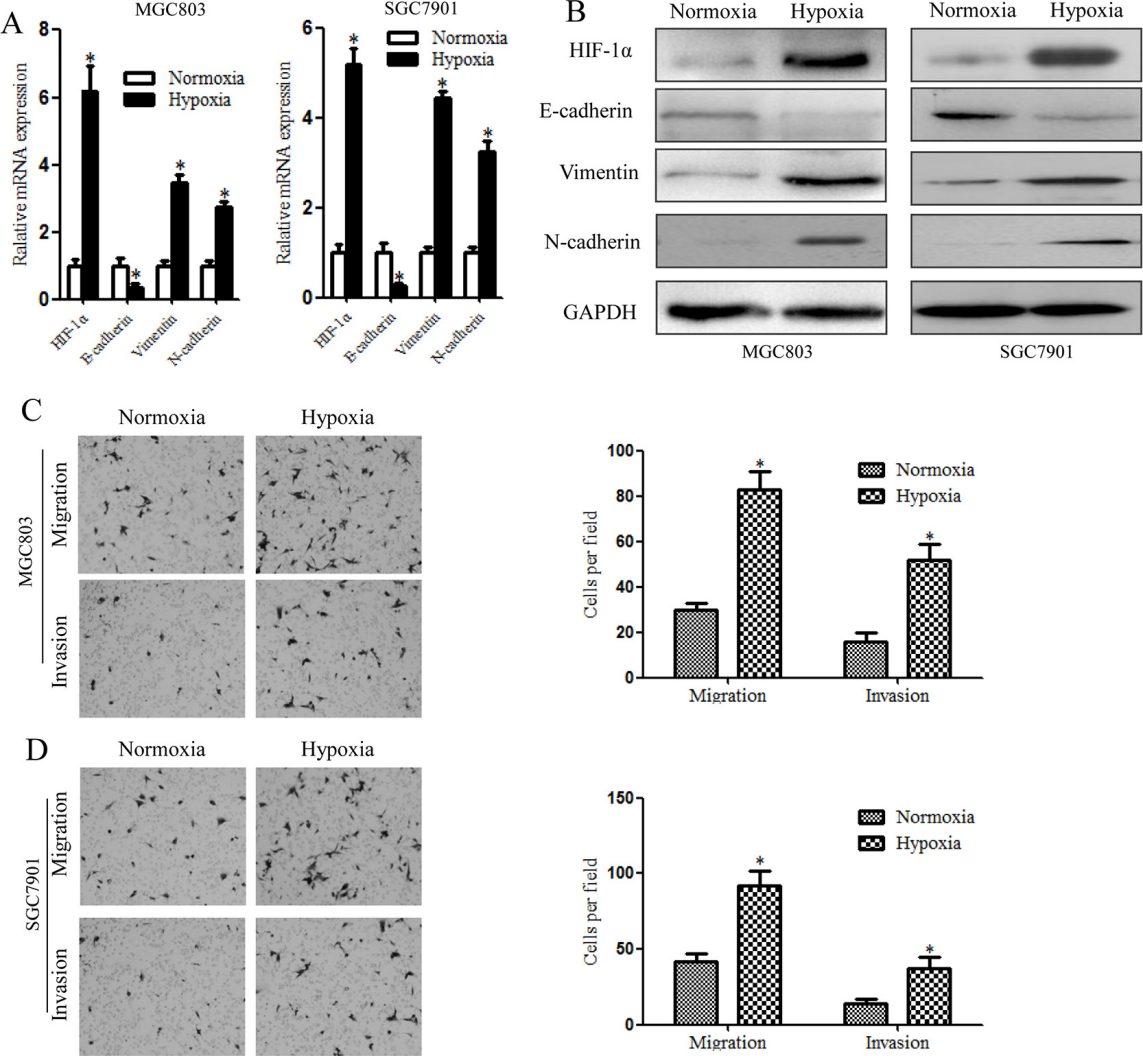
Hypoxia-induced EMT in gastric cancer stem cells, as illustrated by higher levels of EMT-related molecules and increased cell invasiveness (**A**) qRT-PCR showing significantly enhanced EMT-related gene expression in MGC803 and SGC7901 spheroid cells compared with cells under normoxic conditions. (**B**) EMT–related proteins detected by western blotting. Increased HIF-1α, E-cadherin and Vimentin protein levels and decreased E-cadherin protein levels were detected in MGC803 and SGC7901 spheroid cells after 48 h under hypoxic conditions. (**C**) Migration and invasion assay results showed that hypoxia-treated MGC803 spheroid cells exhibit increased migration and invasiveness compared with normoxia-treated cells. The Figures on the right show the quantitative analysis of migrating and invading cells (*P < 0.05). (**D**) Migration and invasion assay of hypoxia-treated SGC7901 spheroid cells. *P < 0.05.

The relationship between the loss of epithelial characteristics and acquisition of mesenchymal characteristics is associated with poorly differentiated histology and a dismal prognosis. CSCs of gastric cancer cell lines MGC803and SGC7901were enriched and identified via formation of spheroid cells. We examined adherent and spheroid gastric cancer cells, and the results showed that the EMT of cells cultured in spheroids method did not change significantly compared with adherent cells (Supplementary Figure S1).Therefore, we investigated a possible link between the generation of EMT-like CSCs and hypoxia by measuring E-cadherin, Vimentin and N-cadherin expression to evaluate EMT progression. MGC803 and SGC7901cells were incubated with 5% CO_2_ and 1% O_2_balanced with N_2_ gas at 37°C for varioustime periods. In our pre-experiment, we first detected HIF-1α levels in spheroid cells exposed to different concentrations of hypoxia for different periods. We found that HIF-1α expression increased after 48 h of exposure compared with after 24 h (mRNA and protein levels, data not shown). Simultaneously, 1% O_2_ exposure shortened the time necessary to achieve the same effect observed with 3% O_2_. Thus, we selected 48 h of exposure to 1% O_2_ for our experiment. Following exposure to hypoxic conditions or normoxic conditions, qRT-PCR was performed to analyze the levels of E-cadherin, Vimentin, N-cadherin and HIF-1α mRNA expression. The results showed that HIF-1α expression increased significantly after hypoxia treatment. In addition, the spheroid cells showed increased levels of Vimentin and N-cadherin and decreased levels of E-cadherin after hypoxia treatment (Figure 4A). Western blotting was performed to confirm this alteration, with the spheroid cells treated with hypoxia exhibiting decreased levels of E-cadherin and increased levels of Vimentin and N-cadherin (Figure 4B). To determine whether these hypoxia-induced EMT-like CSCs have a greater migration and invasive abilities compared to normal CSCs, migration and invasion assays were performed. Hypoxia significantly increased the migration and invasion abilities of MGC803 and SGC7901 CSCs compared with normoxic conditions (Figure 4C, 4D). These data indicate that hypoxia may induce the generation of EMT-like CSCs.

### HIF-1α induced by hypoxia correlates with the generation of EMT-like CSCs and Snail activation

**Figure 5:**
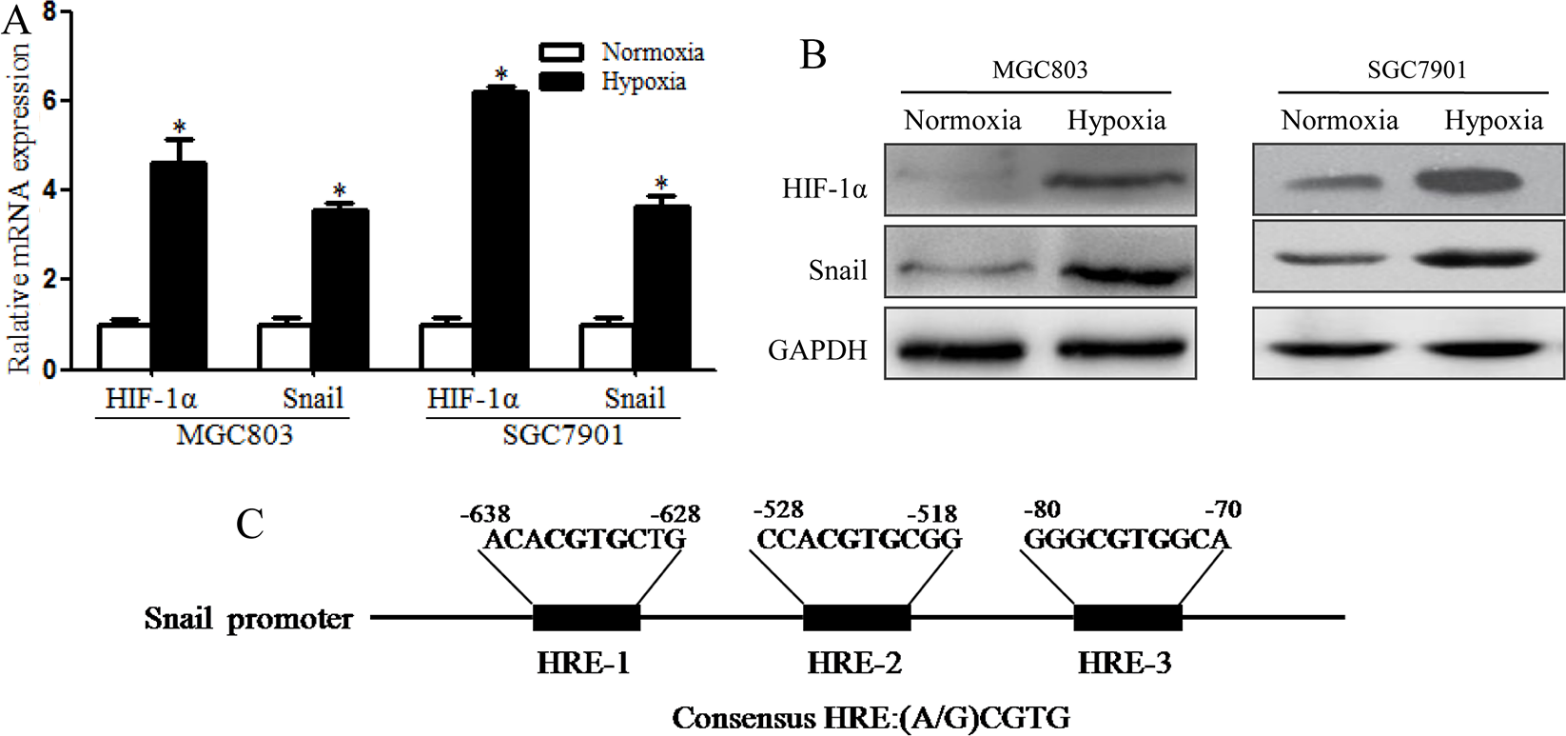
Snail expression was induced by HIF-1α in hypoxia-induced MGC803 and SGC7901 spheroid cells (**A**) RT-PCR analysis showing that mRNA levels of HIF-1α and Snail increased significantly in MGC803 and SGC7901 spheroid cells under hypoxic conditions (*P < 0.05). (**B**) Western blotting showing that HIF-1α and Snail protein levels in MGC803 and SGC7901 spheroid cells increased significantly under hypoxic conditions. (**C**) Different potential HREs in the promoter sequence of the Snail gene and hypoxia response elements (HREs) containing the consensus sequence (A/G) CGTG. The experiment was performed in triplicate and repeated three times. Error bars indicate SD.

Previous studies have shown that hypoxia has an important impact on the CSC microenvironment [12]. Our study showed that EMT-like CSCs can be induced by HIF-1α and that these EMT-like CSCs may be vital to metastasis. Some EMT-related transcription factors, including TCF3, ZEB1, ZEB2, ID2, SNAI2, Notch1 and Twist, which can be induced by hypoxia, may be involved in this process. According to our qRT-PCR results, Snail was the gene most affected (Supplementary Figure S2). Snail is a zinc-finger transcription factor with important functions in tumors; in particular, its expression correlates with reduced cell adhesion and increased cell migration and invasion [15]. Snail is also associated with biological aggressiveness in several tumor types, which promotes EMT induction in this phase [17]. We assert that the function of Snail in the generation of EMT-like CSCs in cancers should be examined because Snail is a crucial inducer that contributes to tumor EMT. Thus, we sought to explore whether enhanced expression of Snail during EMT in CSCs is induced by hypoxia. MGC803 and SGC7901 cells were cultured to form tumor spheres, which were exposed to hypoxic or normoxic conditions for 48 h, followed by qRT-PCR and western blotting for HIF-1α and Snail. The results indicated that hypoxia induced HIF-1α and Snail expression in MGC803 and SGC7901 spheroid cells (Figure 5A, 5B). To investigate whether Snail expression increased significantly via enhanced HIF-1α levels after exposure to hypoxia, we searched for potential HIF-1α binding sites in the Snail promoter and found putative hypoxia response elements (HREs) (Figure 5C). These data provide evidence that Snail expression is increased by hypoxia-induced HIF-1α in gastric CSCs during EMT.

### Suppressing Snail inhibitsHIF-1α-induced EMT-like CSC formation under hypoxia

**Figure 6:**
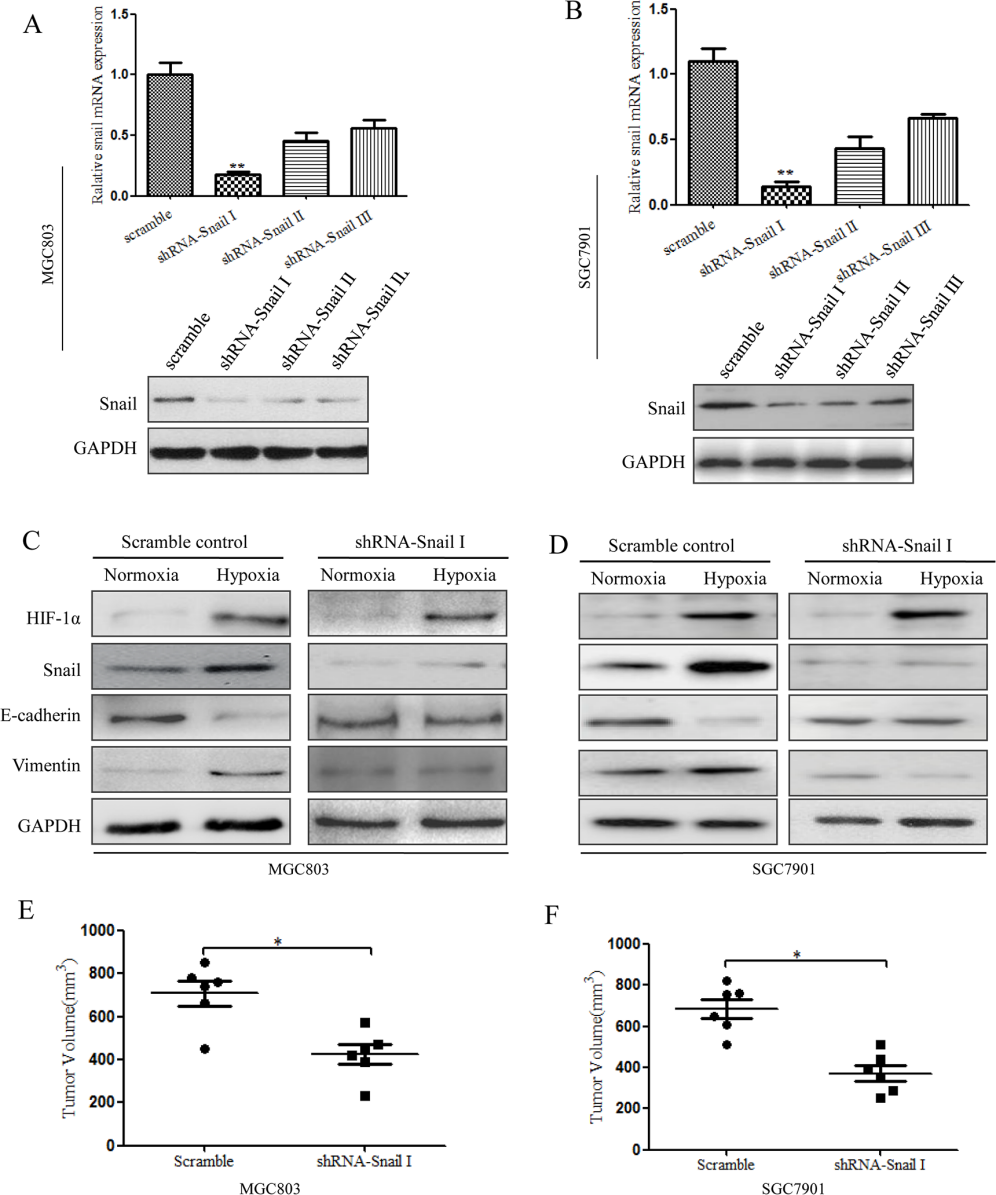
Inhibition of Snail signaling under hypoxia can suppress HIF-1α-induced EMT in CSCs (**A**) qRT-PCR and western blot analysis showing the efficiency of Snail knockdown compared to that of the scrambled control in MGC803 spheroid cells (*P < 0.05). (**B**) qRT-PCR and western blot analysis showing the efficiency of Snail knockdown compared to that of the scrambled control in SGC7901 spheroid cells.(**C**) Western blot analysis showing that Snail shRNA in MGC803 spheroid cells can suppress HIF-1α-induced EMT under hypoxic conditions. The results show no clear changes in the levels of E-cadherin and Vimentin expression. (**D**) Western blot analysis showing that Snail shRNA in SGC7901 spheroid cells can suppress HIF-1α-induced EMT under hypoxic conditions. The experiment was performed in triplicate and repeated three times. (**E**) The effect on tumor volume in nude mice of shRNA-Snail MGC803 spheroid cells after hypoxia induction. *n* = 6 mice, *P < 0.05. (**F**) The effect on tumor volume in nude mice of shRNA-Snail SGC7901 spheroid cells after hypoxia induction. *n* = 6 mice, *P < 0.05.

Our results showed that Snail could be activated by HIF-1α, which is consistent with previous studies [13]. Snail can also repress E-cadherin expression and facilitate invasion and metastasis in some human cancers [18]. To confirm that Snail expression during EMT in gastric CSCs is increased by hypoxia-induced HIF-1α, the expression of Snail was suppressed by specific shRNAs in tumor spheres cultured from MGC803 and SGC7901 cells. qRT-PCR and western blot analyses revealed the efficiency of Snail knockdown at the mRNA and protein levels compared with the scrambled control. The greatest knockdown efficiency was observed with shRNA-Snail I (Figure 6A, 6B). MGC803 and SGC7901 cells transfected with shRNA-Snail I were cultured in CSC medium to obtain Snail-knockdown spheroid cells. We exposed these cells to hypoxic conditions for 48 h, and the changes in EMT and levels of HIF-1α were determined and compared with the scrambled control. The levels of HIF-1α in the two groups increased significantly after hypoxia treatment; however, EMT changes were significantly impaired in Snail-shRNA spheroid cells, and changes in the levels of E-cadherin and Vimentin were not apparent. Conversely, in MGC803 cells, the scrambled control resulted in obvious EMT changes (Figure 6C). Moreover, in SGC7901 cells, knockdown of Snail under hypoxia significantly abrogated the activity of HIF-1α in response to the induction of EMT-like CSCs (Figure 6D). Furthermore, Snail-shRNA MGC803 and SGC7901 spheroid cells displayed much small tumor volumes compared with control cells (Figure 6E, 6F). These findings suggest that Snail knockdown is responsible for reductions in HIF-1α-induced EMT-like CSC generation.

## DISCUSSION

The cancer stem cell theory hypothesizes that cancer stem cells (CSCs), which possess self-renewal capacities and other stem cell properties such as resistance to chemotherapy and radiotherapy, are responsible for tumorigenesis, recurrence and metastasis [2]. We applied the spheroid colony formation method to obtain CSCs from the gastric cancer cell lines MGC803 and SGC7901. Our results indicate that spheroid cells overexpress the CSC-related genes Oct4 and Nanog; this overexpression is a vital characteristic of CSCs. Furthermore, MGC803 and SGC7901 spheroid cells exhibited high invasion capabilities and increased tumorigenicity compared with the parental cells. Our experiments confirmed MGC803 and SGC7901 spheroid cells to been riched for CSCs.

Currently, EMT is considered an essential step in cancer progression and metastasis because it allows cells to migrate, invade surrounding tissues, and escape into the blood stream, suchthat primary tumors can metastasize to other organs [8]. Brabletz found that a subset of CSCs exhibit high invasion and migration capabilities via acquisition of stemness through EMT. Because EMT isassociated with invasive and migratory capabilities, this subset was named migrating CSCs or EMT-like CSCs. However, little is known to date about the regulatory mechanisms of these cells. EMT may be promoted by an inflammatory immune response and by the external environment of atumor [19]. Some environmental factors, such as hypoxia, are also involved in the process of EMT during malignant cell transformation [20]. After exposure to hypoxia, many cancer cells demonstrate increased expression of hypoxia-inducible factor (HIF). The heterodimeric transcription factor HIF-1 is composed of subunitsHIF-1α and HIF-1β.The former is the most important factor induced by hypoxia because HIF-1α controls the expression of target genes containing hypoxic response elements (HREs) in their promoters. Thus, HIF-1α, along with its signaling pathway, is vital for tumor invasion and metastasis [11]. Some transcription factors, such as Snail, are involved in the process of EMT during the malignant transformation of cells. Snail mutation abolishes cell migration because the cells are unable to undergo EMT [13]. In addition, Snail may be regulated by hypoxia in cells with malignant alterations, and a possible link between EMT-like CSCs and hypoxia has been suggested.

In our study, MGC803 and SGC7901 spheroid cells exposed to hypoxia showed enhanced EMT. According to our study, HIF-1α may be involved in promoting EMT. Expression of both HIF-1α and Snail increases during this process, initiating a cascade of events that leads to the changes characteristic of EMT, including decreased E-cadherin expression, increased Vimentin expression and enhanced invasion ability. These findings correspond to the concept of the ‘EMT-like cancer stem cell’ proposed by Brabletz, where by a subset of CSCs exhibit high invasive and migratory capabilities via acquisition of stemness through EMT. Our analyses indicate that hypoxia can induce CSCs to migrate via EMT.

Loss of E-cadherin expression is a fundamental event in EMT, and several previous studies have suggested that Snail binds to and represses activity of the E-cadherin promoter, which is considered an important step in EMT. [21]An HRE may also be present in the Snail promoter; thus, Snail may be induced by HIF-1α [13]. As few studies addressing the relationship between EMT and hypoxia-induced CSCs have been published [20], we explored whether Snail is involved in HIF-1α-induced EMT in CSCs by employing spheroid cells transfected with Snail shRNA. The results showed significant suppression of EMT changes after treatment of these cells with hypoxia; however, changes in E-cadherin and Vimentin expression were not apparent. Nevertheless, the scrambled control still exhibited HIF-1α-induced EMT. Our data indicate that HIF-1α induced acquisition of an EMT phenotype in the spheroid cells, which could be reversed by inhibiting Snail mRNA expression. This finding suggests that Snail knockdown is responsible for the observed differences in HIF-1α-induced EMT among CSCs, which exemplifies the influence of hypoxia on the plasticity of the CSC state. Significant correlations between many important EMT drivers, such as Snail, and cancer patient relapse and survival due to metastasis have been reported, indicating that EMT leads to poor clinical outcomes [22]. EMT-like CSCs may be responsible for tumor metastasis through acquisition of migration and invasion capabilities.

Because Snail can bind to E-box consensus sequences in the E-cadherin promoter with the help of local chromatin structure modifications, leading to repression of E-cadherin expression, we believe that translation of Snail mRNA was strengthened by hypoxia-induced HIF-1αin our study. Such modifications include phosphorylation by PAK and GSK3β, dephosphorylation by small C-terminal domain phosphatase (SCP), and lysine oxidation by LOXL2 [23]. Therefore, hypoxia-induced Snail may promote an EMT phenotype in a subset of CSCs, which subsequently become EMT-like CSCs. Some research has also distinguished non-EMT(CD44^high^ESA^high^) and EMT (CD44^high^ESA^low^)CSCs in squamous cell carcinoma and indicated that two biologically distinct CSC phenotypes have different functions in tumor behavior. For instance, EMT CSCs may dominate with regard to invasion and metastasis [24]. Although much work is needed to fully understand there lationship between EMT and CSCs, we agree with the concept of ‘migrating CSCs’ proposed by Brabletz, which suggests that CSCs can acquire an EMT phenotype or accelerate the growth of surrounding cells with an existing EMT phenotype by some mechanism.

In summary, hypoxia treatment can promote an EMT phenotype in CSCs. Our results demonstrate that hypoxia treatment induces the generation of EMT-like CSCs, which is dependent on the HIF-1α-Snail-EMT axis. Snail is expressed byHIF-1α activation and thus initiates or accelerates EMT in CSCs, possibly resulting in tumor recurrence and metastasis. Our findings represent a potentially new target for therapeutic strategies for gastric cancer.

## MATERIALS AND METHODS

### Cell culture (normoxia and hypoxia)

The human gastric cancer cell lines MGC803 andSGC7901 and HEK293T cells used in this study were purchased from Cell Resource Center of Institutes for Biological Science, Shanghai, China. All cells were cultured in DMEM/F12 medium supplemented with 10% fetal bovine serum (Invitrogen,Carlsbad, USA), 100 U/ml penicillin and 100 mg/ml streptomycin (Gibco, USA)with 5% CO_2_ at 37°C.For hypoxic conditions, MGC-803 and SGC7901cells were incubated with 5% CO_2_ and 1% O_2_ balanced with N_2_ gas at 37°C for the indicated time periods.

### Formation of spheroid cells

Adherent cells were harvested by trypsin digestion; single cells were collected and resuspended in CSC medium to culture spheroid cells. The CSC medium was supplemented with DMEM/F12 (Invitrogen, USA), recombinant human epidermal growth factor (EGF, 20 ng/ml, PeproTech, USA), recombinant human basic fibroblast growth factor (bFGF, 20 ng/ml, PeproTech, USA) and B27 supplement (1X, Invitrogen, USA). The cells were then seeded in ultra-low-attachment 100-mm dishes (Corning, USA) and incubated at 37°C with 5% CO_2_. To assess self-renewal ability, the tumor spheres were gently dissociated with a pipette every 5 d to avoid apoptosis within the spheres. The tumor spheres were dissociated, diluted in CSC medium, and cultured in 96-well ultra-low-attachment dishes (Corning, USA) at a density of a single cell per well.

### Colony formation assay

To analyze differences in colony formation between the spheroid cells and parental cells, the two types of gastric cancer cells were thoroughly dissociated and then plated in 6-well plates at a density of 500 cells per well in triplicate. The cells were cultured in DMEM/F12 with 10% FBS at 37°C with5% CO_2_. After 2 weeks, most cell clones had generated more than 50 cells, which were washed twice with PBS, fixed in methanol for 15 min, and stained with Giemsa dye for 15 min at room temperature. The numbers of colonies that contained more than 50 cells were then counted. This procedure was repeated three times.

### Immunofluorescence staining

Cells were placed on poly-L-lysine-coated glass coverslips and cultured in DMEM/F12 with 10% FBS at 37°C with 5% CO_2_. The cells were fixed with 4% paraformaldehyde for 20 min and washed with PBS; 0.1% Triton X-100 was used to permeabilize the cells, and 0.5% BSA was used as a blocking agent. After washing with PBS, the cells were incubated with a rabbit anti-human Nanog antibody (1:500, Abcam, USA) overnight at 4°C. After washing with PBST, the cells were incubated with a secondary antibody (Alexa Fluor^®^ 488 Goat Anti-Rabbit IgG, 1:200, Invitrogen, USA) for 30 min; nuclei were stained with DAPI (CWBIO, China) for 5 min. The images were visualized by fluorescence microscopy (Olympus, Japan).

### *In vivo* tumorigenicity experiments

The experimental procedures were conducted in compliance with relevant guidelines and regulations of the Animal Ethics Committee of the Third Military Medical University (TMMU), ChongQing, China. The protocols were also approved by the Animal Ethics Committee of TMMU. Four-week-old male BALB/c nude mice were purchased from Beijing Laboratory Animal Center. MGC-803and SGC7901 spheroid cells and parental cells were collected and washed with serum-free HBSS, and then 1 × 10^4^, 1 × 10^5^ and 1 × 10^6^ freshly dissociated cells (uncombined spheroid and parental cells) were suspended in 200 μl DMEM/F12 medium. The spheroid cells or parental cells were injected subcutaneously into each mouse in the appropriate group (6 mice per group). Tumor growth was observed every week; necropsy and final tumor growth assessment were performed for each mouse at 8 weeks after cell implantation. Tumor tissues were fixed in formaldehyde and examined after hematoxylin and eosin staining.

### Migration and invasion assays

For migration and invasion assays, Transwell chambers (BD Falcon, USA) were coated with Matrigel (BD, USA). The Matrigel-coated Transwell chambers were hydrated for at least 2 h in an incubator with 500 μl serum-free DMEM/F12 in the bottom of the well and with 200 μl in the top of the chamber. After the Matrigel was hydrated, the medium in the bottom of the well was replaced with DMEM/F12 plus10% FBS. In total, 2.5 × 10^4^ cells per well were plated in the top of the chamber with 500 μl DMEM/F12 medium and incubated for 24 h at 37°C. After incubation, the cells on the top of the filter were removed with a cotton-tipped swab. The migrating cells were fixed in formaldehyde, stained with Giemsa and counted under an inverted microscope. Migrating cells in five fields of each filter were counted, and we used the average number to represent the number of migrating cells per field.

### Quantitative real-time PCR

Cells were collected and washed three times with phosphate-buffered saline (PBS) pre-chilled at 4°C. Total RNA was extracted using the TRIzol reagent (TaKaRa, Japan) according to the manufacturer's instructions. To generate cDNA, reverse transcription of 1 μg total RNA was performed using a synthesis system (TaKaRa, Japan).The resulting cDNA was subjected to quantitative real-time PCR using the CFX96 Real-Time Quantitative PCR system (Bio-Rad, USA) according to the manufacturer's instructions with SYBR Green I (TaKaRa, Japan).GAPDH was used as an internal control. Data were analyzed by the ΔΔCt method using CFX Manager software(Bio-Rad, USA). A melting curve analysis was performed to ensure the amplification of a single PCR product. Reactions with no template were included as negative controls. Relative quantitation of target gene expression was evaluated using the comparative Ct method. Each qPCR reaction was performed in triplicate. All PCR primers used in this study are shown in Supplementary Table S1.

### Western blotting

Western blotting was used for protein detection. Cells were collected by centrifugation (spheroid cells) or by tryps in digestion (parental cells) and then washed three times with PBS pre-chilled at 4°C.Total cell proteins were extracted using a Tissue Protein Extraction Kit (CWBIO, China) and analyzed by western blotting. The lysates were centrifuged at 12000 rpm for 15 min at 4°C, and the supernatants were collected. Fifty micrograms of each protein sample was loaded onto 8% SDS–PAGE gels and subjected to electrophoresis under denaturing conditions followed by transfer to PVDF membranes (Millipore). The membranes were blocked with 5% BSA in TBST for 2 h at room temperature and then incubated with a rabbit anti-human E-cadherin (1:400, Abcam, USA), rabbit anti-human Vimentin (1:400, Abcam, USA), mouse anti-human N-cadherin (1:400, Abcam, USA), rabbit anti-human Nanog (1:300, Abcam, USA), rabbit anti-human Oct4 (1:300, Abcam, USA), rabbit anti-human HIF-1α (1:500, Abcam, USA), or rabbit anti-human Snail (1:500, Abcam, USA) antibody overnight at 4°C. Rabbit anti-human GAPDH (1:1000, CWBIO, China) was used as an endogenous control. After repeated washes, the membranes were incubated with an HRP-conjugated anti-rabbit or mouse secondary antibody (CWBIO, 1:5000, China). Bands were detected using an enhanced chemilumine scece (ECL) system (Thermo, USA).The membranes were scanned using Bio-Rad GelDoc XR(BIO-RAD, USA), and the images were analyzed using Image Lab software 3.0(BIO-RAD, USA).

### FACS analysis

The two cell types were fixed with 4% paraformaldehyde for 15 min at room temperature and labeled with a mouse anti-human CD133 (1:200, Abcam, USA) antibody for 30 min at 4°C. An Alexa Fluor^®^ 647 goat anti-mouse IgG antibody (1:500, Invitrogen, USA) was subsequently added for 30 min at 4°C. The samples were analyzed by flow cytometry (BD FACSAria II, CA).

### shRNA targeting of Snail mRNA in gastric cancer cells

Snail knockdown in gastric cancer cells was achieved through lentivirus-mediated transduction of Snail mRNA-specific shRNA. Three predesigned target sequences for human Snail were used (Supplementary Table S2). To generate stable transfectants, lentiviral vectors and packaging vectors were cotransfected into HEK293T cells using Lipofectamine(Invitrogen, USA) according to the manufacturer's instructions. Scrambled shRNA was used as a control. Supernatants containing infectious lentiviruses were collected at 24 h after transfection. MGC803 and SGC7901cells were infected with the lentivirus-producing shRNA directed against Snail, and stable transfectants were selected using puromycin for 7 d.

### Statistical analysis

All data are expressed as the mean ± standard deviation. The data were analyzed using SPSS version 15 software. Significant differences between the two groups were analyzed using an independent *t*-test. *P* values less than 0.05 were considered significant.

## SUPPLEMENTARY MATERIALS FIGURES AND TABLES


