# Supplementary material for: HIF-1α induces the epithelial-mesenchymal transition in gastric cancer stem cells through the Snail pathway
Source: Oncotarget. 2017 Jan 4;8(6):9535–45. doi: 10.18632/oncotarget.14484 (PMC5354751; doi:10.18632/oncotarget.14484)
Supplement: Supplementary file 1 [file oncotarget-08-9535-s001.pdf]

HIF-1α induces the epithelial-mesenchymal transition in gastric cancer stem cells through the Snail pathway

Supplementary Materials

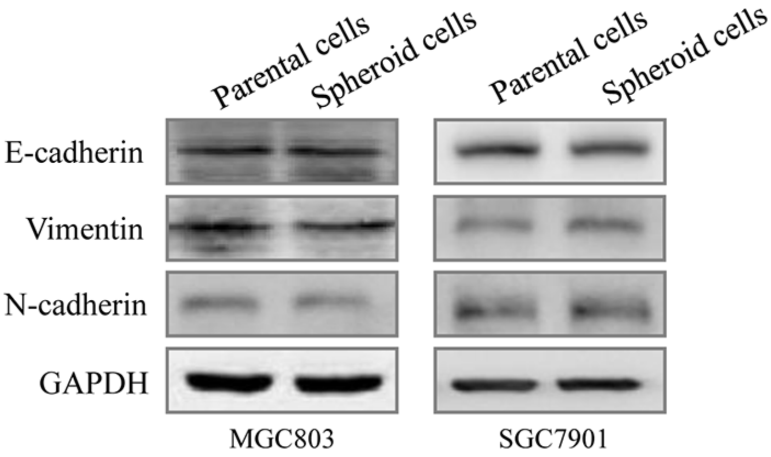

Supplementary Figure S1: The EMT–related proteins in MGC803 and SGC7901 spheroid cells compared with parental cells.

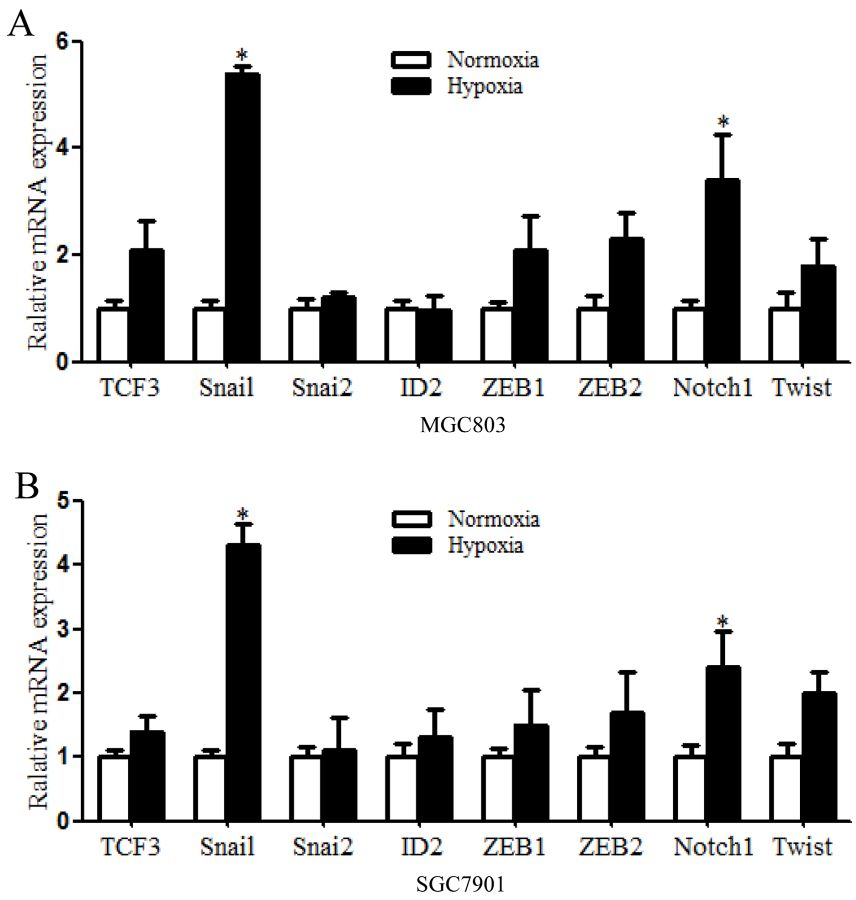

Supplementary Figure S2: Levels of the Snail gene are significantly increased in MGC803 and SGC7901 spheroid cells under hypoxia induction. (**A**) qRT-PCR analysis of Snail, TCF3, ZEB1, ZEB2, ID2, SNAI2, NOTCH1 and TWIST gene expression in MGC803 spheroid cells under hypoxia induction compared with normoxia-treated cells. (**B**) qRT-PCR analysis of Snail, TCF3, ZEB1, ZEB2, ID2, SNAI2, NOTCH1 and TWIST gene expression in SGC7901 spheroid cells under hypoxia induction compared with normoxia-treated cells.

Supplementary Table S1: Sequences of primers used for qRT-PCR in this study

Gene	Forward	Reverse
HIF-1a	5′- GTCGGACAGCCTCACCAAACAGAGC-3′	5′- GTTAACTTGATCCAAAGCTCTGAG-3′
E-cadherin	5′- GGAGGCCTTGGGATACTTTGAA-3′	5′- GAGCTATAGAGGCCTGGGGATTAC-3′
Vimentin	5′- CCAGGCAAAGCAGGAGTC-3′	5′- GGGTATCAACCAGAGGGAGT-3′
NANOG	5′- TGAACCTCAGCTACAAACAG-3′	5′- TGGTGGTAGGAAGAGTAAAG-3′
OCT-4	5′- AGCGAACCAGTATCGAGAAC-3′	5′- TTACAGAACCACACTCGGAC-3′
Snail	5′- CCTCCCTGTCAGATGAGGAC-3′	5′- CCAGGCTGAGGTATTCCTTG-3′
GAPDH	5′- GGCATCCTGGGCTACACT-3′	5′- CCACCACCCTGTTGCTGT-3′
TCF3	5′- CCCAGGAGCCTGAGCTG-3′	5′- CTGTCACCAACGGGAAGG-3′
ID2	5′- TCAGCACTTAAAAGATTCCGTG-3′	5′- GACAGCAAAGCACTGTGTGG-3′
SNAI2	5′- TGACCTGTCTGCAAATGCTC-3′	5′- CAGACCCTGGTTGCTTCAA-3′
NOTCH1	5′- GTTGGGGTCCTGGCATC-3′	5′- GGTGAGACCTGCCTGAATG-3′
TWIST	5′- TCCATTTTCTCCTTCTCTGGAA-3′	5′- GTCCGCGTCCCACTAGC-3′
ZEB1	5′- TGCAGTTTGTCTTCATCATCTG-3′	5′- CCAGGTGTAAGCGCAGAAA-3′
ZEB2	5′- TCCCAATTCTGGAGTAAAGTCTC-3′	5′- GCCACAAATGAAAGTCCTGG-3′

Supplementary Table S2: Sequences of shRNA target to snail in this study

shRNAs	shRNA sequence
shRNA I Forward oligo	5′-CCGGGCAACAAGGAATACCTCAGCCCTCGAGGGCTGAGGTATTCCTTGTTGCTTTTTG-3′
shRNA I Reverse oligo	5′-AATTCAAAAAGCAACAAGGAATACCTCAGCCCTCGAGGGCTGAGGTATTCCTTGTTGC-3′
shRNA II Forward oligo	5′-CCGGCCCACTCAGATGTCAAGAACTCGAGTTCTTGACATCTGAGTGGGTTTTTG-3′
shRNA II Reverse oligo	5′-AATTCAAAAACCCACTCAGATGTCAAGAACTCGAGTTCTTGACATCTGAGTGGG-3′
shRNA III Forward oligo	5′-CCGGGCTCTTTCCTCGTCAGGAACTCGAGTTCCTGACGAGGAAAGAGCTTTTTG-3′
shRNA III Reverse oligo	5′-AATTCAAAAAGCTCTTTCCTCGTCAGGAACTCGAGTTCCTGACGAGGAAAGAGC-3′
